# A systematic review of mosquito coils and passive emanators: defining recommendations for spatial repellency testing methodologies

**DOI:** 10.1186/1756-3305-5-287

**Published:** 2012-12-07

**Authors:** Sheila B Ogoma, Sarah J Moore, Marta F Maia

**Affiliations:** 1 Environmental Thematic Group, Ifakara Health Institute, P.O. Box 53, Ifakara, Morogoro, United Republic of Tanzania; 2 Department of Disease Control, London School of Hygiene & Tropical Medicine, Keppel Street, London, WC1E 7HT, United Kingdom

**Keywords:** Spatial repellents, Pyrethroids, Coils, Passive emanators, Mosquito responses

## Abstract

Mosquito coils, vaporizer mats and emanators confer protection against mosquito bites through the spatial action of emanated vapor or airborne pyrethroid particles. These products dominate the pest control market; therefore, it is vital to characterize mosquito responses elicited by the chemical actives and their potential for disease prevention. The aim of this review was to determine effects of mosquito coils and emanators on mosquito responses that reduce human-vector contact and to propose scientific consensus on terminologies and methodologies used for evaluation of product formats that could contain spatial chemical actives, including indoor residual spraying (IRS), long lasting insecticide treated nets (LLINs) and insecticide treated materials (ITMs). PubMed, (National Centre for Biotechnology Information (NCBI), U.S. National Library of Medicine, NIH), MEDLINE, LILAC, Cochrane library, IBECS and Armed Forces Pest Management Board Literature Retrieval System search engines were used to identify studies of pyrethroid based coils and emanators with key-words “Mosquito coils” “Mosquito emanators” and “Spatial repellents”. It was concluded that there is need to improve statistical reporting of studies, and reach consensus in the methodologies and terminologies used through standardized testing guidelines. Despite differing evaluation methodologies, data showed that coils and emanators induce mortality, deterrence, repellency as well as reduce the ability of mosquitoes to feed on humans. Available data on efficacy outdoors, dose–response relationships and effective distance of coils and emanators is inadequate for developing a target product profile (TPP), which will be required for such chemicals before optimized implementation can occur for maximum benefits in disease control.

## Review

Currently, control of malaria vectors relies almost entirely on indoor residual-spraying (IRS) and long-lasting insecticide-treated nets (LLINs) [1]. These vector control tools have successfully reduced mosquito population densities and malaria by targeting indoor-feeding (endophagic) and indoor-resting (endophilic) mosquitoes [2]. The most successful IRS chemical active used to date is DDT, which, in addition to killing mosquitoes, also reduces indoor mosquito densities consequently reducing malaria transmission [3-6].

Literature shows that much of the success of DDT is due to excito-repellency [4,5]. An excito-repellent is defined as a chemical that causes insects to make undirected movements that set them apart from insecticides [7]. Excito-repellency results from insect’s physical contact with chemicals on treated surfaces or with vapour particles at a distance [8,9]. It has been demonstrated that volatile DDT can induce neural excitement in insects [10] and importantly, it was observed that insects exposed to sub-lethal concentrations of DDT move towards the light explaining why mosquitoes are likely to quickly leave a sprayed dwelling [11]. Excito-repellency was also originally seen as a beneficial feature of pyrethroid treated bednets to reduce the probability of mosquitoes developing resistance to insecticides through lower contact with insecticides [12]. It is known that DDT and pyrethroids act on the voltage-gated sodium channel proteins found in insect nerve cell membranes, disrupting transmission of nerve impulses thereby causing mortality [13]. Cross resistance between DDT and pyrethroids is conferred by point mutations on the voltage gated sodium channel in mosquitoes indicating a common mode of toxic action for these insecticides on mosquitoes [14]. Mechanisms underlying host-seeking and feeding behaviours of mosquitoes are largely unknown and have been the topic of current investigations. It is known that sublethal exposure to both pyrethroids and DDT has a differing effect on insect feeding responses: pyrethroids inhibit responses to attractants while DDT increases neural sensitivity to attractive sources [15,16]. New advancements in the field of neurobiology have demonstrated that perception of chemicals in the environment by insects begins when compounds activate ionotropic receptors, gustatory receptors and olfactory receptors (ORs) located on the dendritic surface of chemosensory neurons of the olfactory receptor cells (ORCs) housed in a head appendage (e.g. antenna or palp) [17]. ORs recognize biologically meaningful chemical ligands, and shape responses of olfactory sensory neurons (OSNs), thus regulating many behaviors including repellency.

Repellents either activate or inhibit action of ORs interfering with the host-seeking behaviour of mosquitoes, resulting in repellency or anti-feeding [18]. A repellent pyrethroid has been shown to disrupt insect behaviour not through targeting the voltage gated sodium channel but instead inhibits the response of odorant receptors (ORs) to attractants in a similar way to para-menthane 3,8 diol and nepetalactone [18]. Repellency is a characteristic of personal protection tools such as mosquito coils, liquid vaporizers, vaporizer mats and ambient emanators [19]. These tools have been extensively studied yet they have not been promoted as formal methods for mosquito control. In 2006 the consumer market for pesticides was about $8.4 billion, with expected double-digit annual growth mainly due to rising income levels in several developing-world markets, notably China [20]. By far the most popular segment was aerosols, at $3.6 billion, followed by topical repellents, powders, and gels at $2 billion. The smaller segments of mats and vaporizers accounted for $1.6 billion and coils for $1 billion [20]. These products are already widely used and would therefore be expected to have community uptake if they were introduced as a formal means of disease control in an integrated vector management (IVM) strategy.

In addition, due to increased need for effective vector control tools, to combat residual outdoor-biting and resting mosquitoes [21], it is timely to review studies of mosquito coils and emanators. This will enable better understanding of their mode of action and hence gain useful knowledge for development of effective spatially acting chemical products that can be used outdoors hence complement LLINs and IRS for integration into a malaria elimination strategy [22].

The main active ingredients recommended by the World Health Organization (WHO) for use in the vapour phase all belong to the pyrethroid chemical class. The most commonly used format; mosquito coils are cheap and effective but produce smoke [23] which is undesirable. Vaporizer mats are an alternative to coils. The mats contain embedded repellent active ingredients that are volatilised using an electric heating element. This need for electricity can increase product costs making them inappropriate for some rural and urban settings in low or middle-income countries.

Recently, other delivery formats that do not require heating or combustion have been developed. These are commonly known as emanators and are composed of insecticides impregnated on substrates such as paper, plastic or agarose-based gels [24,25]. Unlike coils and mats, emanators function through passive evaporation of chemical actives. These chemicals are less polar and have lower vapour pressure than conventional pyrethroids hence evaporate at ambient temperature without the need for an external source of energy. Examples of these insecticides include metofluthrin and transfluthrin.

The aim of this review was to determine effects of mosquito coils and emanators on mosquito responses that reduce human-vector contact and to propose scientific consensus on terminologies and methodologies used for evaluation of product formats that could contain spatial repellents including IRS, LLINs and insecticide treated materials (ITMs).

This review was conducted in accordance with PRISMA (Preferred Reporting Items for Systematic Reviews and Meta-Analyses) guidelines [26]. PubMed, (National Center for Biotechnology Information (NCBI), National Library of Medicine, NIH), MEDLINE, LILACS, Cochrane library, IBECS and Armed Forces Pest Management Board Literature Retrieval System were searched systematically for both field and laboratory studies that included pyrethroid based coils and/or emanators using the English key-words “Mosquito coils”, “Mosquito emanators” and “Spatial repellents”, between January and November 2011. In addition to journal articles, we searched reference lists of identified papers. We also checked the System for Information on Grey Literature in Europe (SIGLE) for unpublished data from sources such as conference proceedings, abstracts and there with ensured that there was no publication bias. The last search was conducted on 21^st^ September 2012. We were confident that the search engines we used provided almost all relevant studies of interest. Data were extracted from selected articles that met all study criteria using a standardized spreadsheet. The information collected included first author, year of publication, methods and design, active ingredient, dose, mosquito species, sample size, description of the control, testing conditions (experimental huts, rooms, chambers or cylinders) and the outcome measures reported with any available statistical information.

### Inclusion and exclusion criteria

**Figure 1:**
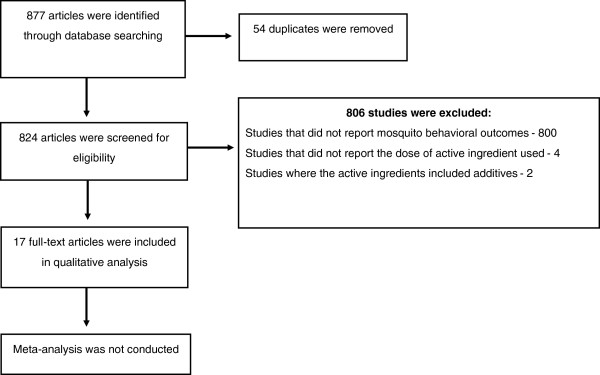
A flow diagram of the selection procedure used for the systematic review of accessible articles.

All publications evaluating coils and/or emanators were reviewed. However, to facilitate comparison of bioefficacy of different active ingredients across studies, the following selection scheme was employed (Figure 1): (i) laboratory and field studies were reviewed separately; (ii) only laboratory and field studies that quantified mosquito responses including biting/feeding inhibition of mosquitoes, knock-down time and percentage mortality 24 hours post-exposure to insecticides, deterrence, repellency or irritancy of insecticides were included; (iii) studies where the dose of active ingredient was not indicated were excluded, (iv) all studies where coils contained a mixture of insecticides or additives were excluded.

### Summaries of reported mosquito responses to coils and emanators, and suggestions for harmonization of terminologies

Several investigators report a number of mosquito responses to airborne insecticide particles. These responses are classified into measurable indicators namely: deterrence, repellency and irritancy, biting/feeding inhibition, knock-down and mortality. Scientific discussions differentiate between mechanisms in mosquitoes leading to responses elicited in the presence of chemical actives and the outcomes quantified [4,7,8,11], this review is restricted to measured behavioural endpoints or consequences and not possible mechanisms causing them.

All studies identified and included in the review evaluated formulated/optimized emanators and coils. It should be noted that comparison of pyrethrins to metofluthrin emanators is only appropriate if both actives were formulated or both were neat material (unoptimized) as effects on volatization and longevity (among other chemical properties) will be different and bias analyses. This holds true even for comparing results of the same active ingredient.

#### Deterrence

Airborne insecticide particles present inside and around houses create a chemical barrier that prevents mosquitoes from entering [27]. Deterrence has been measured in the field by comparing the number of mosquitoes entering houses with insecticides and those without. Coils containing pyrethrins deter between 45% and 80% mosquitoes (Table 1) and 200mg optimized metofluthrin emanators reduce mosquitoes by > 80% within the first 4 weeks of treatment [28]. However, results from these studies cannot be generalized for other spatial repellent compounds due to potential differences in product formulation i.e., optimized components for release and retention. Only one study measured dose-dependent effects of pyrethrum coils [29] and showed no correlation between the proportion of mosquitoes deterred and the dose of pyrethrum (Table 1). Reduced indoor density of mosquitoes in insecticide treated houses could be due to the spatial action of chemical actives which interfere with the host seeking process of mosquitoes making the houses less attractive even when humans are present. In addition, mosquitoes entering treated houses are prevented from feeding. Such observations warrant further investigations of spatially acting chemicals.

**Table 1:** Mosquito behavioral reactions induced by burning coils in experimental huts

**Active ingredient**	**Dose (w/w %)**	**Vector**	**Feeding inhibition (%)**	**Non-Contact irritancy (%)**	**Deterrence (%)**	**Mortality (%)**	**Reference**
Pyrethrum	0.10%	*Anopheles gambiae* Gillies^a^	54	82	51	16	[29]
Pyrethrum	0.10%	*Culex fatigans*	26	58	64	4	[29]
Pyrethrum	0.10%	*Mansonia uniformis*	24	93	45	3	[29]
Pyrethrum	0.50%	*Anopheles gambiae* Gillies	60	87	58	15	[29]
Pyrethrum	0.50%	*Culex fatigans*	46	67	51	7	[29]
Pyrethrum	0.50%	*Mansonia uniformis*	69	87	58	15	[29]

^a^ The sub species of *Anopheles gambiae* Gillies was not specified.

#### Repellency and irritancy

Repellency was originally defined to refer to the distribution of insects/mosquitoes on chemically treated surfaces compared to untreated surfaces [11]. This description considers the end result of the effect of chemicals and does not account for a series of preceding behaviours exhibited by mosquitoes that lead to the final outcome. Therefore, this definition was refined to refer to movement of mosquitoes away from a source to which they would otherwise be attracted [30]. Dethier described two kinds of behaviour causing insects to sit apart from insecticide treated surfaces: [7] “taxis”: - immediate directional reaction, resulting in movement away from a treated surface and; 2) “orthokinesis”: - increased undirected activity after contact with insecticides. Both reactions reduce mosquitoes on treated surfaces [7,8]. These terms have been developed further to include "contact irritancy” where mosquitoes make oriented movement away from a chemical source after physical contact with insecticide treated surfaces [3,4] and “non-contact irritancy”, where mosquitoes move away when exposed to vapour insecticide particles usually operating at a distance. This has also been described as “spatial repellency” [4,31], or “area repellency” [32] or “non-contact disengagement” [8]. Non-contact irritancy, spatial repellency and non-contact disengagement all describe behavioural endpoints resulting from exposure to chemical emanations from coils and emanators. For purposes of clarity we propose that spatial repellency should be used as a general term to refer to the sum of mosquito behaviours produced by airborne chemicals that result in mosquitoes sitting apart from a source of stimulation [8].

“Non-contact irritancy” was measured in the field using local houses or experimental huts fitted with exit- and entry-traps [29,33-35] by comparing the proportion of mosquitoes exiting untreated and treated structures. Using this approach, studies have demonstrated an increased proportion of mosquitoes that exit earlier from huts with burning coils compared to huts which do not have coils [29]. There was a positive correlation between the proportion of mosquitoes exiting huts and the concentration of the active ingredient [29]. This indicates that the magnitude of irritancy might be dose-dependent [31]. An effective way of measuring “non-contact irritancy” is by releasing laboratory-reared mosquitoes inside experimental huts [4] and observing how fast they leave treated huts compared to control huts. This field data demonstrated good correlation with laboratory data from a high-throughput screening system (HiTSS) developed for evaluating behavioural mode of action of active ingredients [4].

#### Biting/feeding inhibition

Feeding or biting inhibition is where mosquitoes are prevented from biting or feeding on humans. Coils reduce the biting rate of mosquitoes (Table 1). Small amounts of insecticides [36] or repellents have been shown to interfere with the host-seeking process of disease vectors [37,38]. Sometimes mosquitoes land on the host but do not feed in the presence of repellent actives [39]. Therefore, the act of feeding (probing) should be quantified rather than landing rate. Only one study displayed an increase in the proportion of mosquitoes inhibited from feeding when the dose was increased [29]. In some cases even the smoke which does not contain chemicals reduces biting rate significantly compared to controls where coils are not used [40]. This warrants the need to conduct more studies with different doses of spatial chemical actives and to generate dose–response curves which will enhance better understanding of the mode of action.

The most accurate and representative method to measure feeding inhibition is through human landing catch (HLC) [41]. Some studies use guinea-pigs as bait [42], which are not proxy indicators for man. A study comparing biting inhibition on guinea pigs and man indicated that guinea pigs underestimated reduction in biting inhibition [42]. This is because guinea pigs do not produce sufficient heat, moisture and carbon dioxide and have a different composition of head space kairomones hence do not attract anthropophilic mosquitoes as much as humans. We propose conducting HLC evaluations inside semi-field systems (SFS) using laboratory reared disease-free mosquitoes to reflect the end use of spatial repellents, while protecting participants from potential exposure to disease carrying mosquitoes.

#### Knock-down and mortality

Knocked-down (KD) is the incapacitation of mosquitoes after contact with a sub-lethal dose of insecticide [43] resulting in the inability of the insect to maintain normal posture or fly.

High concentrations of pyrethrins induce faster KD50 (within 3–5 minutes of exposure) followed by high mortality rate while low concentrations induce slower KD50 (more than 10 minutes after exposure) indicating a dose–response relationship (Table 2). It is also important to note that coils induce up to 95% mortality in laboratory-assays compared to very low levels observed in field-assays (3%–16%) (Table 2). This is attributed to volume and/or ventilation limitations that may occur in some laboratory assay spaces, which reduce insecticide dispersion consequently increasing relative insecticide concentration.

**Table 2:** Knock-down time and mortality of mosquitoes after exposure to smoke from smoldering coils

**Active ingredient**	**Dose (w/w %)**	**Vector**	**Mortality (%)**	**Knock-down (KT50 minutes)**	**Method**	**Reference**
Allethrin	0.60%	*Culex pipiens pallens*	0.12	5.1	70 cm^3^ Chamber	[44]
Allethrin	0.60%	*Stegomyia (Aedes) aegypti*	0.72	3.1	70 cm^3^ Chamber	[44]
Allethrin	0.60%	*Anopheles stephensi*	0.81	3.2	70 cm^3^ Chamber	[44]
Allethrin	0.50%	*Anopheles stephensi*	33	9.5	25m^3^ room	[42]
Allethrin	0.25%	*Anopheles stephensi*	38	11.1	25^3^ room	[42]
Allethrin	0.20%	*Anopheles stephensi*	25	11.3	25^3^ room	[42]
Allethrin	0.15%	*Anopheles stephensi*	32	14.5	25^3^ room	[42]
Allethrin	0.50%	*Stegomyia (Aedes) aegypti*	88	14.9	25^3^ room	[42]
Allethrin	0.25%	*Stegomyia (Aedes) aegypti*	70	24.8	25^3^ room	[42]
Allethrin	0.20%	*Stegomyia (Aedes) aegypti*	54	29.0	25^3^ room	[42]
Allethrin	0.20%	*Anopheles stephensi*	49	4.5	500 mm by 300 mm cylinder	[42]
Allethrin	0.15%	*Anopheles stephensi*	49	4.9	500 mm by 300 mm cylinder	[42]
Allethrin	0.10%	*Anopheles stephensi*	42	5.5	500 mm by 300 mm cylinder	[42]
Allethrin	0.05%	*Anopheles stephensi*	32	6.8	500 mm by 300 mm cylinder	[42]
Allethrin	0.20%	*Stegomyia (Aedes) aegypti*	95	6.2	500 mm by 300 mm cylinder	[42]
Allethrin	0.15%	*Stegomyia (Aedes) aegypti*	73	7.5	500 mm by 300 mm cylinder	[42]
Allethrin	0.10%	*Stegomyia (Aedes) aegypti*	54	10.0	500 mm by 300 mm cylinder	[42]
Allethrin	0.05%	*Stegomyia (Aedes) aegypti*	26	16.0	500 mm by 300 mm cylinder	[42]
d- allethrin	0.30%	*Culex pipiens pallens*	0.15	3.8	70 cm^3^ Chamber	[44]
d- allethrin	2.00%	*Stegomyia (Aedes) aegypti*	0.316	1.57	2m^3^ Peet-Grady chamber	[45]
d- allethrin	2.00%	*Culex quinquefasciatus*	0.491	0.98	2m^3^ Peet-Grady chamber	[45]
d- allethrin	2.00%	*Anopheles stephensi*	0.674	1.94	2m^3^ Peet-Grady chamber	[45]
d- allethrin	0.30%	*Anopheles stephensi*	0.81	2.4	70 cm^3^ Chamber	[44]
d- allethrin	0.30%	*Stegomyia (Aedes) aegypti*	0.84	2.4	70 cm^3^ Chamber	[44]
d,d-T-plarethrin	0.10%	*Stegomyia (Aedes) aegypti*	0.22	171	25m^3^ room	[46]
d,d-T-plarethrin	0.10%	*Stegomyia (Aedes) aegypti*	0.24	120	25m^3^ room	[46]
d,d-T-plarethrin	0.10%	*Culex pipiens quinquefasciatus*	0.25	108	25m^3^ room	[46]
d,d-T-plarethrin	0.15%	*Stegomyia (Aedes) aegypti*	0.25	140	25m^3^ room	[46]
d,d-T-plarethrin	0.20%	*Stegomyia (Aedes) aegypti*	0.28	130	25m^3^ room	[46]
d,d-T-plarethrin	0.10%	*Culex pipiens quinquefasciatus*	0.3	55	25m^3^ room	[46]
d,d-T-plarethrin	0.15%	*Stegomyia (Aedes) aegypti*	0.3	100	25m^3^ room	[46]
d,d-T-plarethrin	0.20%	*Stegomyia (Aedes) aegypti*	0.3	85	25m^3^ room	[46]
d,d-T-plarethrin	0.10%	*Culex pipiens pallens*	0.36	20.6	25m^3^ room	[46]
d,d-T-plarethrin	0.15%	*Culex pipiens pallens*	0.39	14	25m^3^ room	[46]
d,d-T-plarethrin	0.15%	*Culex pipiens quinquefasciatus*	0.47	100	25m^3^ room	[46]
d,d-T-plarethrin	0.20%	*Culex pipiens quinquefasciatus*	0.5	63	25m^3^ room	[46]
d,d-T-plarethrin	0.10%	*Culex pipiens pallens*	0.51	14.5	25m^3^ room	[46]
d,d-T-plarethrin	0.15%	*Culex pipiens pallens*	0.53	11.4	25m^3^ room	[46]
d,d-T-plarethrin	0.15%	*Culex pipiens quinquefasciatus*	0.55	42	25m^3^ room	[46]
d,d-T-plarethrin	0.20%	*Culex pipiens pallens*	0.67	13.1	25m^3^ room	[46]
d,d-T-plarethrin	0.20%	*Culex pipiens quinquefasciatus*	0.71	24	25m^3^ room	[46]
d,d-T-plarethrin	0.10%	*Anopheles dirus*	0.91	8	25m^3^ room	[46]
d,d-T-plarethrin	0.10%	*Anopheles dirus*	0.91	8	25m^3^ room	[46]
d,d-T-plarethrin	0.20%	*Culex pipiens pallens*	0.92	10.3	25m^3^ room	[46]
d,d-T-plarethrin	0.20%	*Anopheles dirus*	1	8.1	25m^3^ room	[46]
dl,d-T80 allethrin	0.27%	*Culex pipiens quinquefasciatus*	0.04	196	25m^3^ room	[46]
dl,d-T80 allethrin	0.27%	*Stegomyia (Aedes) aegypti*	0.15	361	25m^3^ room	[46]
dl,d-T80 allethrin	0.27%	*Culex pipiens pallens*	0.2	28.3	25m^3^ room	[46]
dl,d-T80 allethrin	0.27%	*Stegomyia (Aedes) aegypti*	0.21	174	25m^3^ room	[46]
dl,d-T80 allethrin	0.27%	*Culex pipiens pallens*	0.27	18.6	25m^3^ room	[46]
dl,d-T80 allethrin	0.50%	*Culex pipiens pallens*	0.28	20.8	25m^3^ room	[46]
dl,d-T80 allethrin	0.50%	*Stegomyia (Aedes) aegypti*	0.29	170	25m^3^ room	[46]
dl,d-T80 allethrin	0.27%	*Culex pipiens quinquefasciatus*	0.35	41	25m^3^ room	[46]
dl,d-T80 allethrin	0.50%	*Culex pipiens quinquefasciatus*	0.55	72	25m^3^ room	[46]
dl,d-T80 allethrin	0.27%	*Anopheles dirus*	1	11.1	25m^3^ room	[46]
dl,d-T80 allethrin	0.50%	*Anopheles dirus*	1	8	25m^3^ room	[46]
d-trans allethrin	0.30%	*Culex pipiens pallens*	0.18	3.9	70 cm^3^ Chamber	[44]
d-trans allethrin	0.30%	*Stegomyia (Aedes) aegypti*	0.8	2.5	70 cm^3^ Chamber	[44]
d-trans allethrin	0.30%	*Anopheles stephensi*	1	2.5	70 cm^3^ Chamber	[44]
Esbiothrin	1.00%	*Stegomyia (Aedes) aegypti*	0.301	1.14	2m^3^ Peet-Grady chamber	[45]
Esbiothrin	1.00%	*Culex quinquefasciatus*	0.755	0.81	2m^3^ Peet-Grady chamber	[45]
Esbiothrin	1.00%	*Anopheles stephensi*	0.897	1.68	2m^3^ Peet-Grady chamber	[45]
Pyrethrin	0.30%	*Culex pipiens pallens*	0.12	8.8	70 cm^3^ Chamber	[44]
Pyrethrin	0.30%	*Anopheles stephensi*	0.31	5.2	70 cm^3^ Chamber	[44]
Pyrethrin	0.30%	*Stegomyia (Aedes) aegypti*	0.46	5.5	70 cm^3^ Chamber	[44]
S-d- t -allethrin	0.15%	*Culex pipiens pallens*	0.22	3.6	70 cm^3^ Chamber	[44]
S-d- t -allethrin	0.15%	*Stegomyia (Aedes) aegypti*	0.87	2.5	70 cm^3^ Chamber	[44]
S-d- t -allethrin	0.15%	*Anopheles stephensi*	0.88	2.7	70 cm^3^ Chamber	[44]
Terallethrin	0.15%	*Culex pipiens pallens*	0.38	2.8	70 cm^3^ Chamber	[44]
Terallethrin	0.15%	*Stegomyia (Aedes) aegypti*	0.59	1.8	70 cm^3^ Chamber	[44]
Terallethrin	0.15%	*Anopheles stephensi*	0.73	1.7	70 cm^3^ Chamber	[44]

Optimized metofluthrin emanators induce 100% KD of mosquitoes within 30 minutes of exposure followed by 100% mortality within 24 hours in the laboratory [28]. We did not find any studies that demonstrated correlation between dose and response of mosquitoes to emanators. However, Kawada *et al.* reported that caged mosquitoes placed immediately near metofluthrin-treated paper strips showed 100% KD within 30 minutes and 100% mortality 24-hours post-exposure, while mosquitoes placed 1.5m away from the strip had slower KD and 70% mortality and mosquitoes placed 5m away were unaffected [28]. This could be attributed to decreasing concentration of airborne active ingredients as one moved away from the source. It is noteworthy that these results may not be representative of natural conditions because mosquitoes are confined within the cage thus are likely to take up more active compared to when they are free flying.

The intensity of KD and mortality of mosquitoes is largely dependent on release and degradation rates of actives, initial loading dose on substrate and environmental conditions.

### Harmonization in methodologies for testing spatial mosquito repellents

To characterize behavioural endpoints of mosquitoes exposed to chemical emanations of coils and emanators through rigorous independent and repeatable tests, it is essential to harmonize methodologies used.

#### Mosquito species

The mosquito species selected for bioefficacy studies is dependent on the objective and medical importance of a particular species in a given study area. The World Health Organization (WHO) recommends use of *Stegomyia (Aedes) aegypti* and *Culex quinquefasciatus* for testing household-insecticides [19]. Evaluations should be conducted on both susceptible and resistant strains of different mosquito species. Different mosquito genera, species, and population strains of the same species, vary in their susceptibility to insecticide due to specific selection pressures at site of origin and this can bias the intensity of outcome measures (Table 1). Consequently, we recommend that, when available, mosquito test populations should be acquired from disease endemic areas for which the chemical actives are intended to be used.

#### Size of the laboratory test chambers or rooms

Field and laboratory studies are conducted in chambers, cylinders, rooms or huts of different sizes (Table 2). Mosquitoes are knocked-down faster in cylinders or small chambers compared to large rooms (25m^3^) [42]. This is attributed to low aerial concentration of chemical actives in large ventilated rooms. Peet-Grady chambers [19] are good alternatives to air-tight cylinders. These chambers have improved ventilation provided by built-in fans and a larger volume (180cm by 180cm by180cm) [19]. Tests carried out in Peet-Grady chambers and large rooms demonstrated that KD time was relatively shorter in the chambers than in the rooms [42]. Despite these limitations, cylinders/chambers or small rooms enable precise measurement of mosquito responses to various doses of chemical actives and generation of dose response curves. This might not be possible in field settings where external environmental factors such as wind speed and direction are likely to influence efficacy of the spatially acting actives. Cylinders or small chambers should be used primarily during initial screening for actives. Subsequent studies should then be conducted in more natural environments such as experimental huts and semi-field systems.

#### Environmental factors

The spatial activity of airborne insecticide is dependent on airflow (i.e., air exchange), wind speed, temperature and humidity within the treated space [47]. The greater the air current, the greater the insecticidal dispersion over a specified area followed by reduced insecticide concentration accompanied by dilution of chemical attractants from the human thus reduced host attack by mosquitoes [48]. A study carried out in Tanzania demonstrated reduced efficacy of emanators when used in houses with open eaves [25] compared to houses which did not have eaves in Vietnam [47]. It is necessary to consider the degree of ventilation of the test structure and average environmental conditions during peak disease transmission seasons within the test area where the spatial repellent will be used. High temperature, increases evaporation rate of active ingredient [47] which may improve efficacy but can also lead to faster loss of actives followed by reduced efficacy over time. Therefore, it is necessary to determine the rate at which chemical actives are released from coils and emanators under different environmental conditions in order to determine how much repellent active ingredient will be required for efficacy over time.

#### Experimental design

Other factors affecting experimental outcomes include sample size, which may refer to the number of people used in the trial or number of mosquitoes used and the number of replicates performed during evaluations. It is necessary to determine the number of mosquitoes required for a representative sample. This also applies to the number of human subjects required to account for differences in individual attractiveness to mosquitoes [49-51]. Wherever possible, a balanced Latin Square or William’s Square design with rotation of volunteers and or treatments is desirable. We recommend analysis with generalised linear mixed models [52] which account for over-dispersed nature of repellent mosquito data when variance is greater than mean due to variability caused by the great variability among experimental days [53]. Few of the studies reviewed used appropriate study design or analyses. We propose that future studies report means with standard errors or confidence intervals, or medians with the inter-quartile range in addition to test statistics. This information was not given by most of the studies reviewed, thus, we were unable to conduct a meta-analysis.

## Conclusion

Spatial repellents is the general term used to describe delivery formats such as coils, mats and passive emanators which release vaporised chemical actives capable of affecting mosquito behaviour at a distance. Most vapour chemical actives also knock down, kill or inhibit feeding of mosquitoes. General use of this term causes confusion especially where oriented movement away from the chemical source is not demonstrated. For purposes of clarity we propose that spatial repellency should be used as a general term to refer to sum of mosquito behaviours induced by airborne chemicals that cause mosquitoes to sit apart from a source of stimulation. Despite differences in evaluation methodologies, coils and emanators clearly reduce human-mosquito contact. They induce mortality, deterrence, repellency and reduce feeding of mosquitoes on humans. Mortality was the least observed effect where tests were conducted in experimental huts. This shows that these products do not kill mosquitoes in natural settings with free air movements and therefore may not affect overall mosquito densities or contribute to “community effect” as other toxic insecticides would.

Mosquito coils increased the proportion of mosquitoes exiting huts. It is not clear whether mosquitoes leave treated houses because they are unable to locate hosts for blood meals and therefore continue searching for other blood sources or whether they leave because they are irritated by chemicals in the smoke/vapour and are forced to escape. This needs more investigation.

Reduction in human-vector contact through feeding inhibition is likely to have an epidemiologically significant effect because of reduced risk of getting infectious mosquito bites. Any reduction in human-biting rate of mosquitoes is likely to lower vectorial competence of vectors and affect the lifetime fecundity of vectors which will in turn influence the basic reproductive rate of any parasites that they transmit. In addition to the measure of chemically induced feeding inhibition, it is necessary to conduct studies that quantify fecundity in order to see whether reduced blood feeding consequently reduces fertility of mosquitoes and leads to an overall reduction of mosquito population.

There is minimal data available on dose–response relationships, effective distance and residual efficacy of treated materials. However, the data reviewed here indicate that feeding inhibition, knockdown and mortality are positively influenced by high doses of active ingredient while deterrence does not change with change in dose. However, other studies indicate that deterrence resulting from DDT residues inside huts diminishes with time as the active ingredient degrades [3], indicating a dose dependent-response relationship. Unfortunately, there was no evidence from testing coils and emanators, hence there is need to conduct studies to ascertain this for different doses of coils and emanators under outdoor conditions.

It is hypothesized that since spatial repellents do not kill mosquitoes, there is increased risk of unprotected people being infected with pathogens transmitted by mosquitoes diverted from repellent users [54]. Therefore it is necessary to determine the distance at which non-users are at increased risk of receiving more mosquito bites for repellent-specific actives. On the other hand, non-users may in fact be protected due to airborne dispersion of volatized chemicals. In addition, it is also worthwhile to understand whether feeding inhibition of mosquitoes can be prolonged over several hours or days through product optimization, as this is an epidemiologically significant endpoint for arthropod-borne diseases.

A meta-analysis could not be conducted as a result of the differences in evaluation methodologies as well as minimal statistical parameters reported by various studies. Hence, we strongly underline the need to reach consensus in spatial repellent testing methodologies and data reporting facilitated through the development of standardized assay guidelines. It is important to note that it is highly likely that additional data on spatial repellents has been gathered but not made available to the scientific community. Publication bias due to industry-associated research may contribute to missing data sets, which if shared could greatly contribute to better characterization of spatial repellents. This information is vital for the development of standardized testing methodologies as well as target product profiles. Therefore scientists in industry are encouraged to share their data which will aid this process.

Spatial repellents have the potential to become an important component of vector control since outdoor biting vectors are gaining importance as malaria vectors [55]. In order to understand the dynamics of these products and their potential for vector control programs it is necessary to comprehensively characterize their mode of action (i.e., physiological pathways/receptors and behavioural modification involved in insect response) using standardized methodologies to facilitate the development of a target product profile (TPP) and testing of candidate products so that the required information on their efficacy in disease prevention can be more rapidly collected and policy makers better informed for maximum effective benefit in disease control.

## Competing interests

The authors declare that they have no competing interest.

## Authors’ contribution

SBO conducted the review and drafted the manuscript. SBO, SJM and MFM wrote the final version of the manuscript. All authors read and approved the final manuscript.
